# Efficacy of Xuebijing injection for the treatment of acute lung injury: A meta-analysis of randomized controlled trials

**DOI:** 10.1016/j.heliyon.2024.e33313

**Published:** 2024-06-20

**Authors:** Linfeng Dai, Mingqi Chen, Qiuhua Chen, Yan Zhuang, Hua Jiang, Yong Xu

**Affiliations:** a Department of Critical Care Medicine, Jiangsu Province Hospital of Chinese Medicine, Affiliated Hospital of Nanjing University of Chinese Medicine, Nanjing, China; b School of Chinese Medicine, Nanjing University of Chinese Medicine, Nanjing, China

**Keywords:** Acute lung injury, Xuebijing injection, Meta-analysis

## Abstract

**Background:**

Management guidelines for acute lung injury (ALI) are extremely limited. Xuebijing, a traditional Chinese medicine, exerts therapeutic effects in patients with ALI; however, supportive evidence is currently insufficient.

**Material and methods:**

A systematic literature search of seven electronic databases for randomised controlled trials assessing the efficacy of Xuebijing injections in patients with ALI, published from inception to March 31, 2024, was performed. The Risk of Bias assessment tool recommended by The Cochrane Collaboration was used for quality evaluation. Review Manager version 5.3 (R Foundation for Statistical Computing, Vienna, Austria) was used for analysis. Dichotomous variables are expressed as relative risk (RR) and continuous variables as standardised mean difference (SMD). Heterogeneity was assessed using the *I*^*2*^ statistic and a funnel plot was used to visually assess publication bias.

**Results:**

Sixteen studies comprising 1327 patients were included. Xuebijing injection improved oxygenation index (SMD 1.08 [95 % confidence interval (CI) 0.79–1.38]), reduced the incidence of acute respiratory distress syndrome (RR 0.56 [95 % CI 0.42–0.74) and all-cause mortality (RR 0.48 [95 % CI 0.34–0.67]), and decreased serum tumor necrosis factor-alpha (SMD -1.33 [95 % CI -1.50 to −1.17]) and interleukin-6 levels (SMD -1.35 [95 % CI -1.52 to −1.17]). The funnel plot indicated no publication bias.

**Conclusion:**

Xuebijing injection may be an effective treatment for ALI. However, this needs to be further confirmed in well-designed, large-sample, randomised controlled trials.

## 1 Introduction

Acute lung injury (ALI) is defined as diffuse interstitial and alveolar damage caused by various intrapulmonary and extrapulmonary factors such as severe infection, trauma, aspiration, acute severe pancreatitis, and disseminated intravascular coagulation. ALI refers to a group of clinical syndromes characterised by persistent hypoxaemia (oxygenation index ≤300 mmHg) and even respiratory distress, and exhibits characteristic imaging features, such as exudative shadows, due to increased pulmonary vascular permeability [1]. ALI is estimated to occur in hundreds of thousands of individuals globally each year and, in severe cases, can progress to acute respiratory distress syndrome (ARDS), thereby endangering the lives of affected patients [2]. Nevertheless, the clinical management of ALI is currently extremely limited but centres mainly on active control of the primary disease and symptomatic treatment, such as respiratory support [3].

The pathogenesis of ALI is strongly associated with inflammation, coagulation abnormalities, and alveolar epithelial injury. According to traditional Chinese medicine (TCM), ALI occurs as a result of the joint action and mutual interaction of the accumulation of toxins and internal binding of static blood, and should be treated by detoxifying and eliminating blood stasis. Xuebijing injection is a representative preparation with the effect of removing blood stasis and detoxification, and is commonly used in the clinical treatment of sepsis and severe pneumonia, with good efficacy. Previous studies have revealed that Xuebijing injection suppresses inflammation and alleviates sepsis-induced ALI in animals [[4], [5]]. Recently, several small-sample studies have reported the use of Xuebijing injection for the treatment of ALI. However, extensive evidence-based medical research is lacking, which limits the clinical use of Xuebijing injection for the treatment of ALI. As such, this study comprehensively reviewed the existing relevant literature and systematically evaluated the efficacy of Xuebijing injection in the treatment of patients with ALI through meta-analysis, thus providing a theoretical basis for the use of Xuebijing injection in the clinical treatment of ALI.

## 2 Materials and methods

### 2.1 Literature search

A systematic search of the PubMed, Embase, Web of Science, Cochrane Library, China National Knowledge Infrastructure, Wanfang, and VIP databases was performed for relevant studies published from database inception to March 31, 2024. The search terms used for the Chinese literature were “acute lung injury” AND “Xuebijing”, while those for the English literature were “acute lung injury” OR “ALI” AND “xuebijing”. The reference lists of the retrieved articles on related topics were manually searched for additional, potentially eligible studies.

### 2.2 Literature screening

The inclusion and exclusion criteria were developed according to the Population, Intervention, Comparison, Outcomes, and Study Design principles [6]. The inclusion criteria were as follows (Table 1): randomized controlled trials (RCTs); studies involving patients with a clear diagnosis of ALI of any cause; studies with a control group in which patients were treated with conventional therapy and an experimental group in which patients were treated with Xuebijing injection based on conventional therapy; and studies involving one of the following outcome indicators, incidence of ARDS, all-cause mortality, improvement of oxygenation index, and/or changes in peripheral blood inflammatory indices. The exclusion criteria were as follows: duplicate studies; studies involving a control group in which patients used TCM or Chinese patent medicine preparations other than Xuebijing; and studies with incomplete/unavailable data for meta-analysis.

**Table 1:** Inclusion and exclusion criteria for eligible studies.

Inclusion criteria
Randomized controlled trials;
Studies involving patients with a clear diagnosis of acute lung injury of any cause;
Studies involving a control group in which patients were treated with conventional therapy and an experimental group in which patients were treated with Xuebijing injection based on conventional therapy;
Studies involving one of the following outcome indicators: incidence of ARDS; all-cause mortality; improvement of oxygenation index; and changes in peripheral blood inflammatory indexes
Exclusion criteria
Duplicate studies;
Studies involving a control group in which patients used TCMs or Chinese patent medicine preparation(s) other than Xuebijing;
Studies with incomplete/unavailable data for meta-analysis

ARDS, acute respiratory distress syndrome; TCM, traditional Chinese medicine.

### 2.3 Data extraction and quality assessment of the included literature

Data extraction and quality evaluation of the included studies were performed independently by two researchers. Discrepancies/disagreements between the two researchers were resolved by consensus discussion or adjudicated by a third researcher. Extracted data included author, publication date, interventions in the experimental and control groups, treatment duration, and outcome indicators.

The methodological quality of the included studies was evaluated using the Risk of Bias Assessment tool recommended by The Cochrane Collaboration, including selection, performance, detection, follow-up, reporting, and other biases [7]. Bias results were categorised as high-, low-, or unclear risk.

### 2.4 Statistical methods

Review Manager version 5.3 (R Foundation for Statistical Computing, Vienna, Austria) was used for analysis. Dichotomous variables are expressed as relative risk (RR) and continuous variables as standardised mean difference (SMD). If the heterogeneity between studies was small (*I*^*2*^ ≤ 50 %), a fixed-effects model was used to combine the studies; if the heterogeneity was obvious (*I*^*2*^ > 50 %), a random-effects model was used. For experimental results expressed as *P* values, differences were considered to be statistically significant at *P* < 0.05. Funnel plots were used to evaluate publication bias among the included studies [8]. Subgroup analyses were performed to determine possible sources of bias.

## 3 Results

### 3.1 Search results

**Fig. 1:**
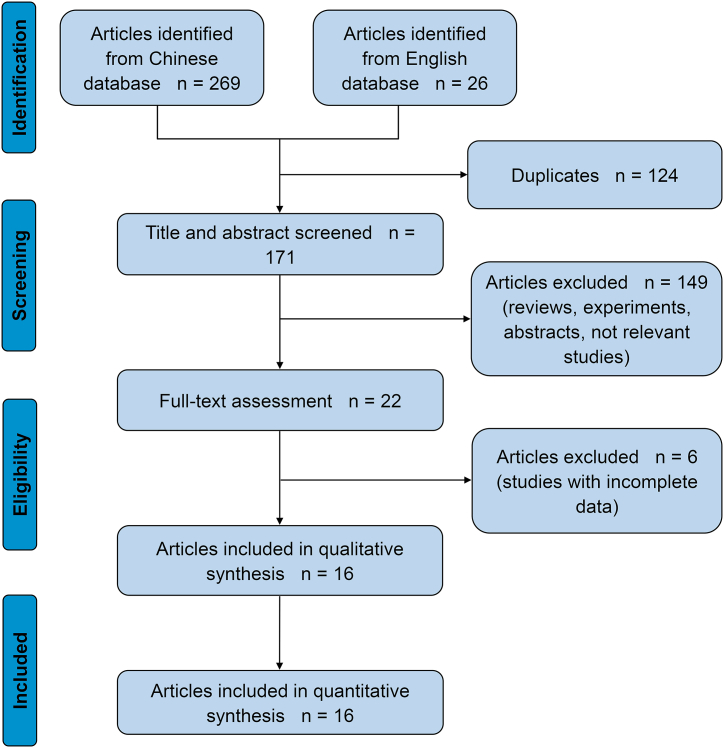
Flow chart of study section.

A total of 295 studies were retrieved in the initial literature search of the aforementioned databases in accordance with the search strategies for the Chinese and English literature. Of these, 124 duplicates were removed, and 22 potentially eligible studies were obtained by assessing the titles and abstracts according to the inclusion and exclusion criteria. After reading the full texts, six studies without complete data for meta-analysis were excluded. Ultimately, 16 studies were included in the present investigation. Details of the literature screening process are illustrated in Fig. 1.

### 3.2 Basic characteristics of the included studies

Sixteen RCTs were included, all of which were conducted nationally and published between 2009 and 2022, with sample sizes ranging from 51 to 144, and a total of 1327 patients [[9], [10], [11], [12], [13], [14], [15], [16], [17], [18], [19], [20], [21], [22], [23], [24]]. In these studies, patients in the control groups were treated with conventional therapy, including correction of triggers, respiratory support, and even glucocorticoids, if necessary. For control patients, Ulinastatin injection was additionally used in two studies [10,21], and Ambroxol hydrochloride injection was additionally used in one study [23]. Patients in the experimental group were additionally treated with Xuebijing injection based on the treatment in the control group. Treatment durations in the studies were distributed as follows: three days (n = 1); 14 days (n = 3); and seven days (n = 12). Characteristics of the included studies are summarised in Table 2.

**Table 2:** Characteristics of included studies.

Author (s)	Publication time	Sample size（T/C）	Duration (day)	Outcomes
Chen et al.[9]	2018	108（54/54）	7	(1)(4)
Gong et al.[10]	2022	120（60/60）	7	(1)
Huang et al.[11]	2021	86（43/43）	7	(4)
Huang et al.[12]	2010	80（40/40）	7	(1)(2)(3)
Liang et al.[13]	2012	70（35/35）	7	(1)(3)(4)
Liu et al.[14]	2020	144（72/72）	7	(1)(3)(4)
Liu et al.[15]	2009	51（27/24）	7	(2)(3)
Luo et al.[16]	2009	71（35/36）	5–7	(3)
Ouyang et al.[17]	2012	60（30/30）	7	(4)
Shen et al.[18]	2017	76（38/38）	14	(1)(3)(4)
Sheng et al.[19]	2015	68（34/34）	7	(1)(2)(3)
Tang et al.[20]	2013	50（25/25）	7	(2)
Wang et al.[21]	2011	60（30/30）	14	(1)(2)(3)
Xiao et al.[22]	2010	63（32/31）	7	(4)
Zhang et al.[23]	2013	62（32/30）	3	(1)(2)
Zhao et al.[24]	2018	80（40/40）	14	(4)

Outcomes: (1) oxygenation index; (2) the incidence of acute respiratory distress syndrome; (3) all-cause mortality; (4) inflammatory factors. T: treatment group; C: control group.

### 3.3 Quality assessment of the included studies

**Fig. 2:**
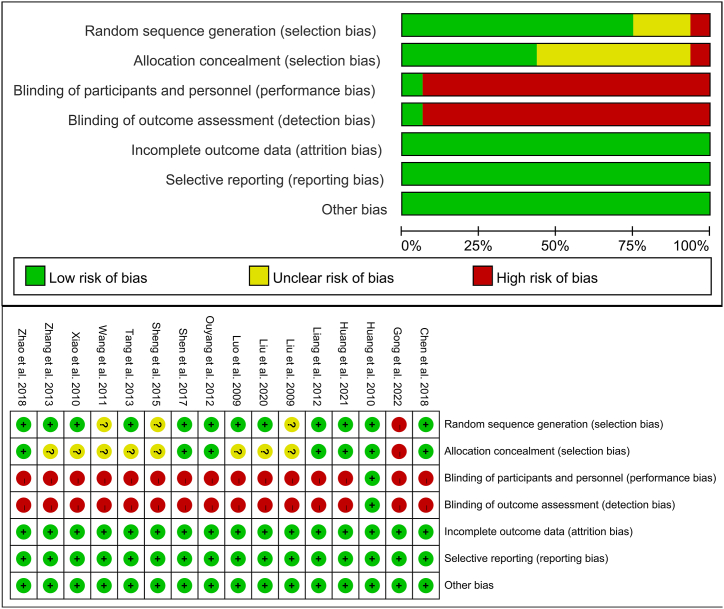
Risk of bias assessment for included studies.

In terms of randomisation methods, 12 studies were rated as low-risk [9,[11], [12], [13], [14],[16], [17], [18],^20^,[22], [23], [24]], 3 as unclear-risk [15,19,21], and 1 as high-risk [10]. With regard to allocation concealment, 7 studies were judged as low risk [9,[11], [12], [13],^17^,^18^,^24^], 8 as unclear risk [[14], [15], [16],[19], [20], [21], [22], [23]], and 1 as high risk [10]. Regarding blinding of participants and researchers, 15 studies were rated as high risk [[9], [10], [11],[13], [14], [15], [16], [17], [18], [19], [20], [21], [22], [23], [24]], and 1 study as low risk [12]. With regard to blinding of study results, 15 studies were judged as high risk [[9], [10], [11],[13], [14], [15], [16], [17], [18], [19], [20], [21], [22], [23], [24]], and 1 as low risk [12]. Regarding incomplete outcome data, selective reporting of outcomes, and other biases, all studies were rated low risk (Fig. 2).

### 3.4 Meta-analysis of outcome indicators

#### 3.4.1 Oxygenation index

**Fig. 3:**
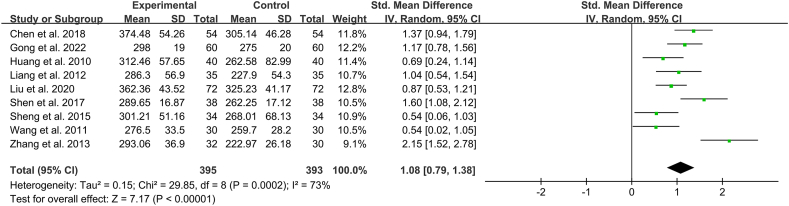
Individual and overall effects of Xuebijing Injection on oxygenation index in patients with acute lung injury.

Nine studies (n = 788) reported improvements in oxygenation index [9,10,[12], [13], [14],^18^,^19^,^21^,^23^]. Due to heterogeneity among these studies (*I*^*2*^ = 73 %), a random-effects model was used to combine the studies, and results of analysis revealed that the improvement in oxygenation index in the Xuebijing injection group was significantly superior to that in the control group (SMD 1.08 [95 % confidence interval (CI) 0.79–1.38]; *P* < 0.00001) (Fig. 3).

Considering the significant heterogeneity across studies, sub-analyses were performed according to possible factors, including treatment duration and cause of ALI. The results of subgroup analyses are summarised in Table 3. The results for each subgroup were consistent with those of the primary analyses. Heterogeneity became non-significant across studies when the treatment duration was 7 days and those in which the cause of ALI was sepsis. However, heterogeneity remained significant in the other subgroups.

**Table 3:** Subgroup analyses for comparation of oxygenation index.

Subgroups	Number of studies	SMD	95 % CI	Heterogeneity
Treatment duration				
3 days	1	2.15	1.52-2.78	/
7 days	6	0.97	0.79-1.14	46 %
14 days	2	1.07	0.03-2.10	88 %
Cause of ALI				
Acute pancreatitis	3	0.94	0.50-1.38	69 %
Sepsis	2	1.32	1.01–1.63	39 %
Trauma	4	1.08	0.45-1.70	83 %

SMD, standardized mean deviation; CI, confidence interval; ALI, acute lung injury.

#### 3.4.2 Incidence of ARDS

**Fig. 4:**
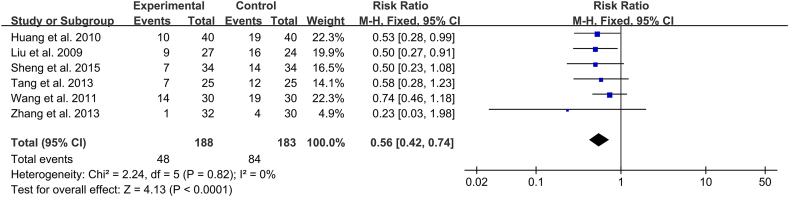
Individual and overall effects of Xuebijing Injection on all-cause mortality in patients with acute lung injury.

Six studies (n = 371) reported the incidence of ARDS, with low heterogeneity (*I*^*2*^ = 0 %) [12,15,[19], [20], [21],^23^]. As such, a fixed-effects model was used to combine the studies, which demonstrated that the incidence of ARDS was statistically and markedly lower in the Xuebijing injection group than in the control group (RR 0.56 [95 % CI 0.42–0.74]; *P* < 0.0001) (Fig. 4).

#### 3.4.3 All-cause mortality

**Fig. 5:**
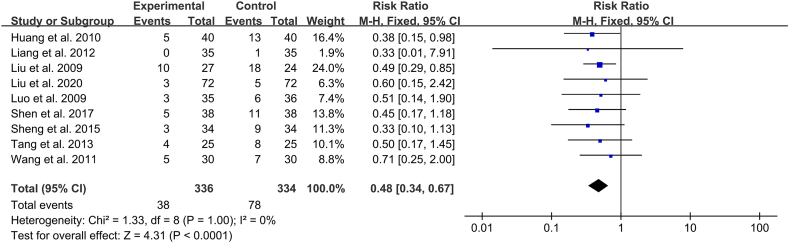
Individual and overall effects of Xuebijing Injection on the incidence of acute respiratory distress syndrome in patients with acute lung injury.

Nine studies (n = 670) analysed all-cause mortality, with low heterogeneity (*I*^*2*^ = 0 %) [[12], [13], [14], [15], [16],[18], [19], [20], [21]]. A fixed-effects model was used to combine the studies, which revealed that the all-cause mortality rate in the Xuebijing injection group was statistically and substantially lower than that in the control group (RR 0.48 [95 % CI 0.34–0.67]; *P* < 0.0001) (Fig. 5).

#### 3.4.4 Inflammatory factors

**Fig. 6:**
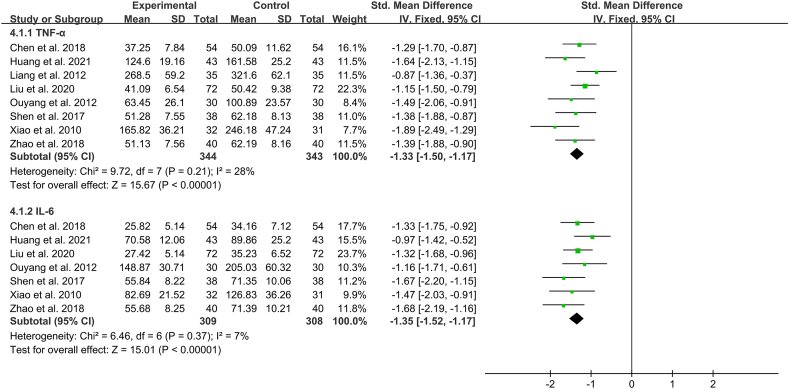
Individual and overall effects of Xuebijing Injection on the improvement of inflammatory factors in patients with acute lung injury.

Eight studies (n = 687) investigated tumor necrosis factor α (TNF-α) levels in peripheral blood [9,11,13,14,17,18,22,24]. Because the heterogeneity among the studies was low (*I*^*2*^ = 28 %), a fixed-effects model was used to combine the studies. Results revealed that TNF-α levels in peripheral blood were statistically and prominently lower in the Xuebijing injection group than in the control group (SMD -1.33 [95 % CI -1.50 to −1.17]; *P* < 0.00001). Seven studies (n = 617) explored interleukin 6 (IL-6) levels in peripheral blood, with an *I*^*2*^ value of 7 % [9,11,14,17,18,22,24]. A fixed-effects model was used to combine the studies, and the results demonstrated that patients in the Xuebijing injection group had significantly lower IL-6 levels in the peripheral blood than those in the control group (SMD -1.35 [95 % CI -1.52 to −1.17]; *P* < 0.00001) (Fig. 6).

### 3.5 Adverse events

Only one study reported adverse events [10]. The incidence of adverse events in the treatment group was 6.67 % (four of 60 patients), whereas that in the control group was 5 % (three of 60 patients); the difference between the groups was not statistically significant (i.e., *P* > 0.05).

### 3.6 Publication bias of the included literature

**Fig. 7:**
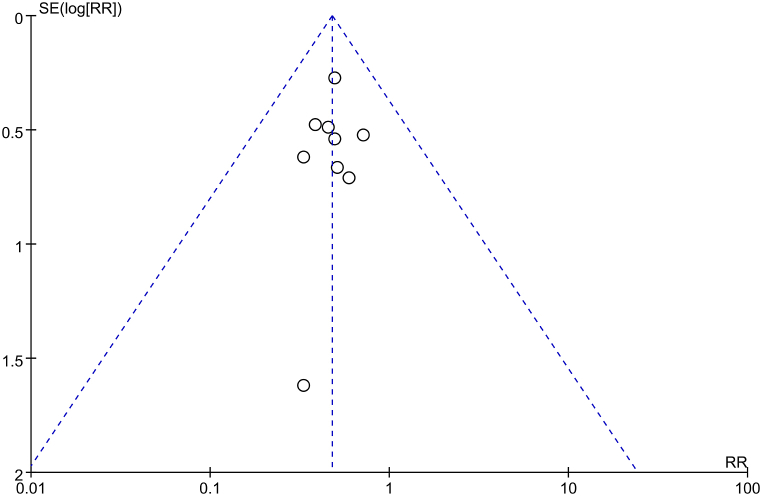
Funnel plot for evaluation of publication bias.

The funnel plot of studies reporting all-cause mortality (Fig. 7) displayed symmetrical scatters on the left and right sides, suggesting no significant publication bias.

## 4 Discussion

The present systematic review and meta-analysis was the first to assess the efficacy of Xuebijing injection in the treatment of ALI, and included 16 RCTs comprising 1327 patients. Results revealed that the additional use of Xuebijing injection was more effective in reducing the levels of peripheral blood inflammatory factors, improving the oxygenation index, decreasing the incidence of ARDS, and significantly lowering mortality in patients with ALI.

Although “ALI” is not specifically recorded in ancient TCM texts, it falls into the categories of “fulminant dyspnea” and “chest binding syndrome” according to its clinical symptoms [25]. An attack by exogenous pathogenic factors is the pathogenic basis of ALI because the lungs occupy the “upper jiao”, with the main function of controlling respiration and, therefore, cannot dominate qi, with impaired diffusion and downbearing, when it is attacked by exogenous pathogenic factors, consequently inducing symptoms such as coughing and shortness of breath. “Stasis-toxin” is an important pathological factor in ALI. “The lungs converge with all vessels and govern management and regulation”, with the role of assisting the heart in promoting blood circulation throughout the body. When an exogenous pathogenic factor enters the lungs and transforms into heat, it can burn the blood vessels so that blood is obstructed and stagnates in the lungs, or trauma damages lung collaterals, causing abnormal flow and accumulation of blood to stagnate, both of which affect the function of the lungs governing diffusion purification and descent [26]. According to modern medicine, ALI pathologically manifests as non-cardiogenic pulmonary oedema, which is attributed to damage to pulmonary capillary endothelial and alveolar epithelial cells. The stimulation of internal and external factors elicits systemic inflammation in the body, which results in the release of a large number of inflammatory factors that increase the permeability of the alveolar-vascular barrier and cause alveolar oedema or even collapse, and the ratio imbalance of ventilation/blood flow, thereby contributing to the occurrence of hypoxaemia. These inflammatory mediators activate the coagulation system and impair the fibrinolytic system, which transforms the intra-alveolar environment into a pro-coagulant and anti-fibrinolytic state, eventually forming a microthrombus in the lungs and leading to pulmonary hypertension [27]. Hence, modern medicine understands that abnormal activation of the coagulation system and excessive inflammation are co-initiating factors of ALI, which is highly compatible with the TCM theory of “stasis-toxin” and provides a theoretical basis for the treatment of ALI by removing stasis and detoxifying.

Xuebijing injection was developed by Prof. Jinda Wang on the basis of the famous formula “*Xuefuzhuyu decoction*” under the guidance of TCM theories of “three syndromes and three methods” and “co-treatment of bacteria, toxins and inflammation”, which consists of *Ligusticum wallichii*, *Salvia miltiorrhiza*, *Carthamus tinctorius*, *Radix Paeoniae Rubra*, and *Angelica sinensis,* and is a representative preparation with the effect of removing blood stasis and detoxifying. Modern pharmacological studies have shown that Xuebijing injection is the main active ingredient in safflower yellow A, ligustrazine, and danshensu, with anti-inflammatory, anti-endotoxic, coagulation regulatory, and anti-oxidative stress effects [28]. Xuebijing injection is often clinically used in the treatment of respiratory, abdominal, and urinary tract infections, and has been approved for the treatment of systemic inflammatory response syndrome in severe and critically ill patients with coronavirus disease 2019 (COVID-19) after the outbreak of the COVID-19 pandemic. In recent years, several well-designed, multicentre, double-blind trials have demonstrated the ability of Xuebijing injection to reduce short-term mortality and improve prognosis in patients with severe pneumonia and sepsis [[29], [30]]. However, the efficacy of Xuebijing injection in the treatment of ALI is unclear and, moreover, has not been the subject of large RCTs. Our meta-analysis provides a comprehensive review of existing small-sample clinical trials and reveals that Xuebijing injection has therapeutic potential in patients with ALI. Meanwhile, our study demonstrated that Xuebijing injection significantly diminished the levels of inflammatory factors, such as TNF-α and IL-6, in the peripheral blood of ALI patients. Considering the important role of inflammation in the progression of ALI, this may be one reason why Xuebijing improved relevant clinical markers. Nevertheless, this hypothesis warrants further investigation. Abnormal coagulation function is considered to be a factor that drives the progression of ALI. However, clinical trials of anticoagulation therapy have not yielded satisfactory results. Therefore, the cross-activation of coagulation and inflammation has been proposed as a possible mechanism of ALI, and bidirectional anti-inflammatory and anticoagulant therapies may be a potential research direction for ALI [31]. As a representative preparation for removing blood stasis and detoxifying, Xuebijing contains *Salvia miltiorrhizae* and safflower, and has good efficacy in activating blood circulation to dissipate blood stasis. However, our meta-analysis failed to incorporate coagulation-related endpoint indicators, which require further study.

The present meta-analysis had several limitations, the first of which were the inclusion of mostly single-centre studies with small sample sizes. The quality of the included studies was low, with no description of allocation concealment and blinding, thus leading to possible performance or detection biases owing to their subjective influence on our results. Second, for the comparison of oxygenation index, the heterogeneity across studies was significant, which could not be satisfactorily explained through the sub-analyses performed for our speculated reasons. Third, the included studies were conducted in China, with some racial biases.

## 5 Conclusions

Currently, the clinical management of ALI mainly involves active control of the primary disease and symptomatic treatment such as respiratory support. Results of the present meta-analysis revealed that Xuebijing injection improved the oxygenation index, decreased the incidence of ARDS, and markedly diminished mortality in patients with ALI, and that its clinical efficacy may be related to the reduction of inflammatory factor levels in the peripheral blood of patients. Overall, Xuebijing may be an effective treatment for ALI. However, this needs to be further confirmed in well-designed, large-sample RCTs.

## Funding

This work was supported by 10.13039/501100001809National Natural Science Foundation of China (81703895).

## Patient consent for publication

Not required.

## Provenance and peer review

Not commissioned; externally peer reviewed.

## Data availability statement

All data relevant to the study are included in the article or uploaded as supplementary information.

## CRediT authorship contribution statement

**Linfeng Dai:** Writing – original draft, Methodology, Investigation, Formal analysis, Data curation. **Mingqi Chen:** Methodology, Investigation, Data curation. **Qiuhua Chen:** Methodology, Investigation, Data curation. **Yan Zhuang:** Formal analysis. **Hua Jiang:** Writing – original draft. **Yong Xu:** Conceptualization.

## Declaration of competing interest

The authors declare that they have no known competing financial interests or personal relationships that could have appeared to influence the work reported in this paper.
